# Integrative genomic analysis for the functional roles of *ITPKC* in bone mineral density

**DOI:** 10.1042/BSR20181481

**Published:** 2018-11-30

**Authors:** Hsing-Fang Lu, Henry Sung-Ching Wong, Ben-Kuen Chen, Hsien-Tzung Liao, Yu-Wen Hsu, Shiro Ikegawa, Er-Chieh Cho, Kuo-Sheng Hung, Wei-Chiao Chang

**Affiliations:** 1 School of Pharmacy, Taipei Medical University, Taipei 11042, Taiwan; 2 Laboratory of Bone and Joint Diseases, RIKEN Center for Integrative Medical Sciences, Tokyo 108–8639, Japan; 3 Master Program for Clinical Pharmacogenomics and Pharmacoproteomics, School of Pharmacy, Taipei Medical University, Taipei 11042, Taiwan; 4 Department of Pharmacology, College of Medicine, National Cheng Kung University, Tainan 70101, Taiwan; 5 Division of Allergy, Immunology and Rheumatology, Department of Internal Medicine, School of Medicine, College of Medicine, Taipei Medical University, Taipei 11042, Taiwan; 6 Division of Allergy, Immunology and Rheumatology, Department of Medicine, Taipei Veterans General Hospital, Taipei, Taiwan; 7 Faculty of Medicine, National Yang-Ming University, Taipei, Taiwan; 8 The Ph.D. Program for Translational Medicine, College of Medical Science and Technology, Taipei Medical University and Academia Sinica, Taipei 11042, Taiwan; 9 Department of Clinical Pharmacy, School of Pharmacy, College of Pharmacy, Taipei Medical University, Taipei 11042, Taiwan; 10 Department of Neurosurgery, Taipei Medical University-Wan Fang Hospital, Taipei 11696, Taiwan; 11 Graduate Institute of Injury Prevention and Control, College of Public Health, Taipei Medical University, Taipei 11042, Taiwan; 12 Department of Pharmacy, Taipei Medical University-Wanfang Hospital, Taipei 11042, Taiwan; 13 Center for Biomarkers and Biotech Drugs, Kaohsiung Medical University, Kaohsiung 80708, Taiwan; 14 PhD Program in Biotechnology Research and Development, College of Pharmacy, Taipei Medical University, Taipei 11042, Taiwan

**Keywords:** ITPKC, osteoporosis, single nucleotide polymorphism

## Abstract

Osteoporosis is defined by low bone mineral density (BMD), which is mainly due to the imbalances in osteoclast and osteoblast activity. Previous studies indicated that early activation of osteoclasts relies on calcium entry through store-operated calcium (SOC) entry, and several genes, including STIM1, ORAI1, and ITPKC, are known as key regulators of SOC entry. However, the relationships between *STIM1, ORAI1, ITPKC*, and human BMD are still unclear. In order to investigate the plausible associations between these genes and BMD, we conducted a meta-analysis of genes expression and BMD using the publicly available GEO database. We further recruited 1044 subjects and tested associations between polymorphisms in these genes and BMD. Clinical information (including age, sex, and BMI) was collected and used for the analysis. Our results indicated that *ITPKC* gene expression was significantly associated with BMD. Furthermore, we found that one *ITPKC* SNP (rs2607420) was significantly associated with lumbar spine BMD. Through bioinformatics analysis, rs2607420 was found to be very likely to participate in the regulation of *ITPKC* expression. Our findings suggest that *ITPKC* is a susceptibility gene for BMD, and rs2607420 may play an important role in the regulation of this gene.

## Introduction

Osteoporosis is a skeletal disorder characterized by low bone mineral density (BMD) that increases the risk of fracture [1]. Its prevalence among the elderly population in Taiwan is 24.15% in females and 11.03% in males [2]. Several factors contribute to the pathogenesis of osteoporosis, including age, sex, and genetics [3]. The heritability of BMD has been estimated at 50–85% according to twin and family studies [4,5], and numerous genes have been identified as being related to osteoporosis and BMD in candidate gene-based studies and genome-wide association studies (GWASs) [6]. The largest meta-analysis of GWASs, published by the GEFOS consortium, revealed several loci associated with BMD [7,8]. Most of the BMD-related common variants function within the RANK–RANKL–OPG signaling pathway, and the low-frequency variants of *EN1* have effects on BMD as well. Based on these and other findings, BMD is widely considered to be a polygenic trait.

Bone loss is due to imbalances between osteoclastic bone resorption and osteoblastic bone production. Store-operated calcium (SOC) entry is an important regulator of osteoclast differentiation [9,10], and when early-stage osteoclasts receive the signals from the receptor activator of the nuclear factor κ-B ligand (RANKL), the store-operated calcium channel will be activated, causing calcium influx [9]. This influx leads to calcium oscillations that activate downstream nuclear factor of activated T cells c1 (NFATc1) to promote osteoclastogenesis [9,11]. The SOC channel is the main calcium influx pathway in non-excitable cells—including T cells, mast cells, and osteoclasts—and it is activated by depletion of calcium stores in the endoplasmic reticulum (ER) [12–14]. Upon depletion of stored calcium in the ER, stromal interacting molecule 1 (STIM1) will aggregate and bind ORAI1 to induce influx of extracellular calcium [15]. Inositol-trisphosphate 3-kinase C (ITPKC) is a negative regulator of the SOC entry, which affects the NFATc1 signaling pathway through the phosphorylation of inositol 1,4,5-trisphosphate (IP_3_) [16,17]. Once IP_3_ is phosphorylated to IP_4_, IP_3_ receptors on the ER are incapable of activating SOC entry, contributing to calcium reductions in the cell [18].

In 2003, Mentaverri et al*.* [19] reported that 2-aminoethoxy-diphenyl borate (2-APB) and SKF-96365, two SOC channel blockers, can significantly decrease bone resorption and the survival of osteoclasts. Additionally, knockdown of *STIM1* expression or inhibition of IP_3_R in bone marrow macrophages reduced calcium signaling and diminished osteoclast differentiation [20]. Hwang et al*.* [21] demonstrated that by using short hairpin (sh)RNA to silence *ORAI1*, a SOC entry component in the plasma membrane, RANKL-induced osteoclastogenesis was blocked. Moreover, compared with wild-type mice, ORAI1^−/−^ mice lacked multinucleated osteoclasts and exhibited markedly decreased cortical ossification and BMD [22,23]. Together, these reports strongly suggest that SOC entry is an important regulatory signal for bone remodeling.

Since the RANKL-induced calcium influx in the early stages of osteoclastogenesis occurs via SOC entry [24], genes in the SOC pathway are likely to be involved in the regulation of bone metabolism. As such, *ITPKC*, an upstream regulator of SOC entry, is a potential candidate gene for osteoclastogenesis as well. However, the genetic relationships between *STIM1, ORAI1, ITPKC*, and BMD remained unclear in humans. In the present study, we aimed to investigate the association between these genes and BMD by examining data from the Gene Expression Omnibus (GEO) database and individual’s genotype from 1044 subjects.

## Methods

### Dataset collection

Gene expression datasets were collected by searching the GEO database (https://www.ncbi.nlm.nih.gov/geo/) with the following key words: ‘osteoporosis’, ‘BMD’, and ‘Homo sapiens’. We included studies in the analysis of their phenotype data that are available. Other diseases as their major phenotype or the study only contained control samples were excluded. For each study, we extracted information including sample number, platform, phenotype, cell type, and gene expression data.

### Meta-analysis of gene expression

Raw expression values of each dataset were normalized using Robust Multi-array Average (RMA) algorithm [25]. If raw CEL files were not available, the processed expression values were downloaded directly form GEO. Bioconductor *hgu133a.db* package was used to annotate each probe ID with its corresponding gene symbol. Each dataset was normalized and mapped to gene symbol individually, and we conducted a meta-analysis by combing expression mean and standard deviation value of each study afterward. R *meta* package was used to perform meta-analysis and generate forest plot. A statistical test of heterogeneity between studies was estimated by *I*^2^. Both the fixed-effect and random-effects model were implemented in this analysis. We used meta-regression under REML model to analyze the high heterogeneity result. The metafor package of R was used to conduct this analysis.

### Patients and methods

We enrolled a total of 1044 patients from Wan-Fang Hospital, Taipei, Taiwan. The study protocol conformed to the Declaration of Helsinki. Males and postmenopausal women aged ≥55 years who visited Neurological Clinics of Wan Fang Hospital due to back pain or lumbar radiculopathy were recruited for the present study. Patients with pathological fractures or high-impact fractures (such as those due to motor vehicle accidents) and continuous steroid use (of over 6 months) were excluded. Patients with the long-term inflammatory disease were also excluded.

BMD was measured by dual energy radiograph absorptiometry with standard protocols at the lumbar spine (LS; L2-4 or L1-4) and femoral neck (FN). Vertebral fractures were assessed by digital measurements of morphologic changes on a lateral radiograph of the thoracolumbar spine. We collected clinical information of the subjects such as age, gender, and BMI. The study was approved by the Joint Institutional Review Board of Taipei Medical University. All subjects provided written informed consent.

### Genotyping

DNA was extracted from whole-blood samples using a Gentra (Qiagen, Valencia, CA) extraction kit and 70% alcohol precipitation. The region we used to select tagging SNPs was defined by the position of each gene locus ± 1500 KB. Based on HapMap SNP database (release27 phase II + III Feb 09, dbSNP b126) and the Haploview 4.2, our study selected tagging SNPs among each linkage disequilibrium (LD) block (*r*^2^ = 0.8). Besides, the SNP with any prior functional report had the priority to be selected. We selected six tagging single-nucleotide polymorphisms (tSNPs) of *ITPKC*, four tSNPs of *STIM1*, and five tSNPs of *ORAI1* with a minimum allele frequency of ≥10% in a Beijing Han Chinese and Taiwanese population (Supplementary Figures S1–3). Genotyping was performed with a TaqMan Allelic Discrimination assay (Applied Biosystems, Foster City, CA). A polymerase chain reaction (PCR) was carried out using an ABI StepOnePlus Thermal Cycler (Applied Biosystems). In a subsequent PCR, the fluorescence from specific probes was detected and analyzed through the System SDS software version 2.2.2 (Applied Biosystems).

**Figure 1:**
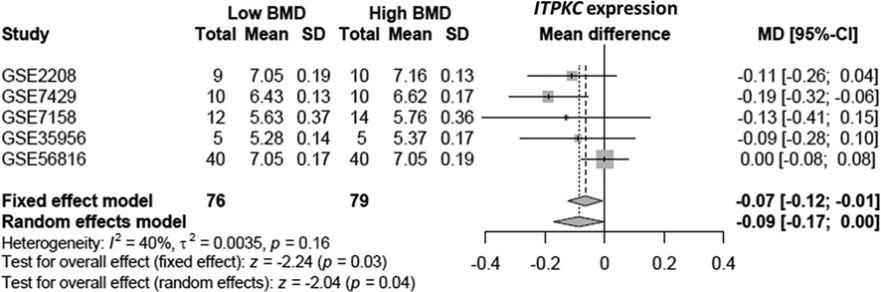
Forest plot of *ITPKC* expression profiling across five datasets

### Functional annotation of SNPs

To investigate the association between gene expression profiles and the SNPs of *ITPKC*, we also queried the GTEx Portal (http://www.gtexportal.org/home/) that includes a variety of tissue expression quantitative trait loci (eQTLs). HaploReg browser (www.broadinstitute.org/mammals/haploreg), which provides regulatory elements estimated by ENCODE and Epigenomics project data, was also used to discover the potential influence of these SNPs.

### Statistical analysis

R 3.2.0 was used for the statistical analyses. Associations between genotypes and BMD at the two sites were tested by the likelihood ratio test. Age, body mass index (BMI), and sex were adjusted in the models as potential confounders. Pairwise LD among genotyped SNPs was assessed and used to define haplotype blocks via Haploview software vers. 4.1 [26]. The general linear model (GLM) implemented in R were used to examine the associations between haplotypes and BMD. To correct for multiple testing, the false discovery rate (FDR) was applied, and *q* values were estimated to control for proper type I errors. FDR *q* values of <0.05 were considered statistically significant.

## Results

### Studies included in the meta-analysis

After carefully curating available data and removing duplicate datasets, five distinct gene expression datasets were downloaded from GEO. Three datasets were based on RNA extracted from peripheral blood monocytes, and the other two datasets were derived from RNA collected from B lymphocytes and mesenchymal stem cells, respectively. Overall, we included 155 individuals from five datasets. Among them, 79 belonged to the high BMD group and 76 belonged to the low BMD group. Most of the subjects were female, except one male in the high BMD group. The detailed parameters of each study are displayed in Table 1.

**Table 1:** Characteristics of the individual studies

Dataset	Race	Sample size (High: Low BMD)	Subjects	Cell	Phenotype	Platform
GSE2208	Caucasian	19 (10:9)	Pre-and postmenopausal female	Monocytes	BMD	GPL96 [HG-U133A]
GSE7429	Caucasian	20 (10:10)	Postmenopausal female	B lymphocytes	BMD	GPL96 [HG-U133A]
GSE7158	Chinese	26 (14:12)	Premenopausal female	Monocyte	BMD	GPL570 [HG-U133_Plus_2]
GSE35956	Caucasian	10 (5:5)	Female and male (9:1)	Mesenchymal stem cells	BMD	GPL570 [HG-U133_Plus_2]
GSE56816	Caucasian	80 (40:40)	Pre- and postmenopausal female	Monocyte	BMD	GPL96 [HG-U133A]

BMD: bone mineral density; HG-U133A: AffymHG-U133_Plus_2etrix Human Genome U133A Array; HG-U133_Plus_2: Affymetrix Human Genome U133 Plus 2.0 Array

### Meta-analysis

Figure 1 shows the forest plot of *ITPKC* expression profiling for the low and high BMD groups across all examined studies. The mean difference in *ITPKC* expression between low BMD and high BMD groups was −0.07 ( 95% CI: −0.12 to −0.01). Based on a fixed effect model, the expression level of *ITPKC* was significantly associated with BMD (*p* = 0.03), even under a random-effects model (*p* = 0.04). There was no severe heterogeneity across studies (*I*^2^ = 40%). Neither *STIM1* nor *ORAI1* showed any significant association between expression level and BMD status (Supplementary Figures S4 and S5). The heterogeneity of *STIM1* was 89%, so we further conducted a meta-regression. This high value was mainly due to differences in cell types, which accounted for 94.93% of the heterogeneity.

### Demographic and clinical characteristics of subjects

A total of 1044 individuals (794 females and 250 males) were recruited in this study (Table 2). The mean age ± standard deviation was 68.5 ± 9.4 years for females and 71.2 ± 9.8 years for males.

**Table 2:** Baseline characteristics of the Taiwanese population

	Female	Male	Total
Number (%)	794 (76.1%)	250 (24.0%)	1044
Age (years)	68.5 ± 9.4	71.2 ± 9.8	69.1 ± 9.6
Body-mass index (kg/m^2^)	25.2 ± 3.9	24.8 ± 3.3	25.1 ± 3.8
Lumbar spine BMD (g/cm^2^)	0.95 ± 0.17	1.11 ± 0.21	0.98 ± 0.19
Femoral neck BMD (g/cm^2^)	0.74 ± 0.12	0.82 ± 0.14	0.76 ± 0.13

Data are presented as the mean ± SD or number (%). BMD, bone mineral density.

### Associations between genetic polymorphisms and BMD of LS and FN

The frequencies of the tag SNPs for *ITPKC* (rs7257602, rs7251246, rs890934, rs10420685, rs2607420, and rs2290692), *STIM1* (rs2304891, rs3750996, rs1561876, and rs3750994), and *ORAI1* (rs12320939, rs12313273, rs7135617, rs6486795, and rs712853) were similar to those from the Taiwan Biobank [27] (Supplementary Table S1). After adjusting for age, sex and BMI, the SNP, rs2607420, was significantly associated with LS BMD (*q* = 0.028). Furthermore, SNP rs10420685 was associated with FN BMD (*p* = 0.028) (Table 3); however, the association did not reach statistical significance after multiple testing corrections (*q* = 0.131). The genetic polymorphisms in *STIM1* and *ORAI1* did not reach statistical significance in either sites (Supplementary Table S2).

**Table 3:** Association between single-nucleotide polymorphisms (SNPs) in *ITPKC* and bone mineral density in the entire population

SNP	Genotype	Lumbar spine					Femoral neck				
		Number	Mean	SE	*p* value	*q* value	Number	Mean	SE	*p* value	*q* value
rs7257602	G/G	175	0.997	0.015	0.300	0.493	208	0.749	0.008	0.387	0.493
	A/G	317	0.965	0.011			377	0.753	0.007		
	A/A	211	0.981	0.013			254	0.763	0.008		
rs7251246	C/C	193	0.968	0.013	0.084	0.214	235	0.759	0.009	0.644	0.644
	C/T	386	0.984	0.010			448	0.76	0.006		
	T/T	213	1.000	0.014			254	0.756	0.007		
rs890934	T/T	169	0.995	0.015	0.107	0.214	202	0.753	0.008	0.350	0.493
	G/T	374	0.991	0.010			436	0.761	0.006		
	G/G	240	0.974	0.012			288	0.764	0.008		
rs10420685	G/G	38	0.993	0.032	0.576	0.620	44	0.782	0.020	**0.028**	0.131
	A/G	252	0.989	0.012			297	0.767	0.007		
	A/A	473	0.981	0.009			559	0.751	0.005		
rs2607420	C/C	61	0.938	0.021	**0.003**	**0.028**	68	0.747	0.014	0.386	0.493
	C/T	288	0.969	0.012			344	0.757	0.008		
	T/T	422	0.997	0.009			499	0.763	0.006		
rs2290692	G/G	181	0.974	0.014	0.070	0.214	219	0.762	0.008	0.460	0.537
	C/G	366	0.982	0.010			429	0.763	0.007		
	C/C	224	1.003	0.014			268	0.752	0.007		

The *p* value was adjusted for age, sex, and the body-mass index. *p* and *q* values of < 0.05 are shown in bold. *q* values of < 0.05 were considered statistically significant after correction for multiple testing.

### Haplotype associations for *ITPKC* and BMD at the LS and FN

**Figure 2:**
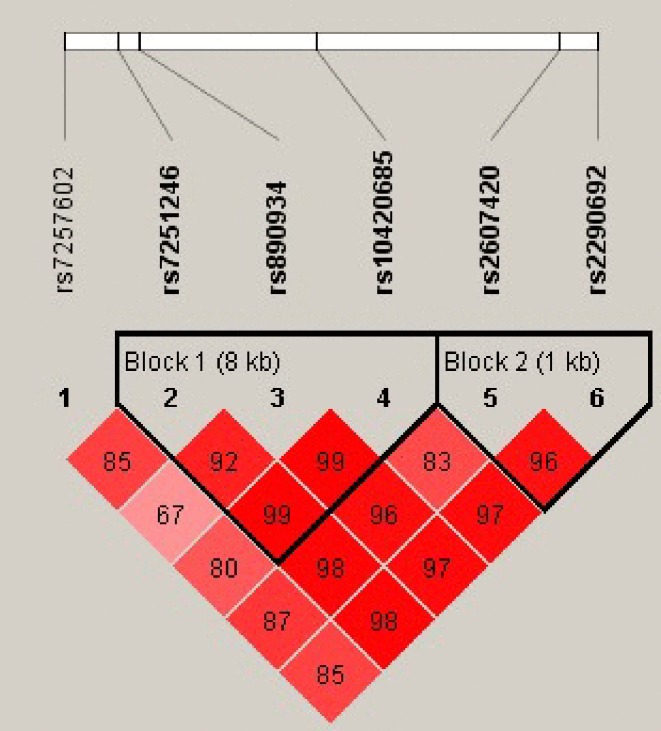
Linkage disequilibrium and haplotype block structure of the *ITPKC* gene The number in a cell is D’ x 100.

In order to elucidate the most important haplotype of *ITPKC*, we further constructed a LD map for *ITPKC*. Two haplotype blocks were found after pair-wise LD analysis of the genotyped SNPs (Figure 2). A haplotype association analysis was then performed on each block. For haplotype block one, formed by rs7251246, rs890934 and rs10420685, the C-G-A haplotype was significantly associated with LS BMD compared with the reference T-T-A haplotype after adjusting for covariates (*p* = 0.008) (Table 4). The C-G haplotype of block two was compared with the T-C haplotype and significant association was found with LS BMD (*p* = 0.008). Neither haplotype block showed a significant association with FN BMD (Table 4).

**Table 4:** Associations of *ITPKC* haplotypes with the bone mineral density

Haplotype	Frequency	Lumbar spine		Femoral neck	
		β	*p* value	β	*p* value
Block 1: rs7251246-rs890934-rs10420685					
TTA	0.435	Reference		Reference	
CGA	0.263	−0.027	**0.008**	−0.005	0.458
CGG	0.213	−0.003	0.800	0.013	0.054
CTA	0.016	0.023	0.480	−0.014	0.515
TGA	0.072	0.002	0.892	0.001	0.950
Block 2: rs2607420-rs2290692					
TC	0.521	Reference		Reference	
CG	0.255	−0.026	**0.008**	−0.001	0.826
TG	0.218	−0.002	0.861	0.012	0.058

β represents the regression coefficient. Haplotype frequency less than 1% was excluded. The analyses were performed under an addictive model adjusted for age, sex, and BMI. Significance (*p* < 0.05) shows in bold.

## Discussion

In the present study, we first conducted a meta-analysis of *STIM1, ORAI1*, and *ITPKC* gene expression based on GEO data. We then further investigated the association between BMD and SNPs in these three genes using 1044 subjects. Our results revealed that among the genes we examined, only *ITPKC* expression was positively correlated with BMD. In addition, one intronic SNPs of *ITPKC*, rs2607420, showed strongly association with LS BMD, yet there was no statistically significant association between polymorphisms of *ITPKC* and FN BMD (Table 4).

Previous studies have illustrated the importance of ORAI1 in bone metabolism. In the present study, we found that the genetic association between *ITPKC* and BMD is more pronounced. This observation may due to the fact that *ITPKC* acts as a negative regulator of the SOC channel, which can directly influence calcium signals. Therefore, our data suggest that either the expression level or genetic polymorphisms of *ITPKC* may be a better biomarker to predict BMD compared with *ORAI1* or *STIM1-*related measurements.

Our results also showed that SNPs may have site-specificity effects, which is consistent with previous studies [7]. Because the proportions of constituent cortical and trabecular bones are distinct at different body locations [28], and various genes differentially regulate the two types of bones, the same genetic polymorphism may have different impacts at different sites [29,30]. Analysis of eQTLs using the GTEx Portal indicated that rs2607420 was significantly associated with the expression of *ITPKC, ADCK4*, and *C19orf54* (Supplementary Table S3). According to 1000 Genomes (Pilot 1 CHB + JPT), rs2607420 is in high LD (*r*^2^ > 0.6) with other SNPs that are located in *C19orf54* and *ADCK4* (Supplementary Figure S6). Moreover, the HaploReg browser indicated that rs2607420 is an intronic SNP, which lies in enhancer histone marks (H3K27ac and H3K9ac) and DNase I-hypersensitivity site in several cell lines including primary T cells and B cells from peripheral blood [31]. These results support a functional role for *ITPKC* in BMD regulation.

A limitation of our study is that we did not collect bone remodeling markers and fracture data to conduct association analyses. These types of data may be required to investigate the further relationships between genetic polymorphisms and osteoporosis-related clinical events. Besides, by using tagging SNP study design we did not genotype all the genetic variants of *ITPKC*. Target sequencing may be used to detect the potential causal variants. Importantly, other replication studies are needed to validate our results. Although several bioinformatics tools have allowed us to understand the potential functions of *ITPKC* polymorphisms in determining the risk of osteoporosis, functional studies on animal models in relevant tissues are still needed to clarify the underlying mechanisms.

## Conclusion

In summary, our results not only revealed a relationship between *ITPKC* expression and BMD, but also identified specific BMD-related loci within the *ITPKC* gene. These results highlight the role of *ITPKC* in determining inter-individual variations in BMD. Our findings may be useful in the development of novel diagnostic tools or treatment targets for osteoporosis in the future.

## Supporting information

**Supplementary Figure 1:** Positions of the tag single nucleotide polymorphisms in the human *ITPKC* gene. Black and white boxes are coding and non-coding regions, respectively.

**Supplementary Figure 2:** Positions of the tag single nucleotide polymorphisms in the human *STIM1* gene. Black and white boxes are coding and non-coding regions, respectively.

**Supplementary Figure 3:** Positions of the tag single nucleotide polymorphisms in the human *Orai1* gene. Black and white boxes are coding and non-coding regions, respectively.

**Supplementary Figure 4:** Forest plot of *STIM1* expression profiling across five datasets.

**Supplementary Figure 5:** Forest plot of *ORAI1* expression profiling across two datasets.

**Supplementary Figure 6:** Regional plot of rs2607420.

**Supplementary Table 1:** Basic characteristics of the tag single-nucleotide polymorphisms (SNPs) in *ITPKC.*

**Supplementary Table 2:** Association between single-nucleotide polymorphisms (SNPs) in *STIM1, ORAI1* and bone mineral density in the entire population.

**Supplementary Table 3:** eQTL results from GTEx

## Abbreviations

BMD: bone mineral density
BP: biological process
eQTL: tissue expression quantitative trait loci
FDR: false discovery rate
FN: femoral neck
GEO: Gene Expression Omnibus
GO: Gene Ontology
ITPKC: Inositol-trisphosphate 3-kinase C
LD: linkage disequilibrium
LS: lumbar spine
SNP: single-nucleotide polymorphism
SOCE: store-operated calcium entry
STIM1: stromal interacting molecule 1
