# Supplementary material for: Integrative genomic analysis for the functional roles of ITPKC in bone mineral density
Source: Biosci Rep. 2018 Nov 30;38(6):BSR20181481. doi: 10.1042/BSR20181481 (PMC6265621; doi:10.1042/BSR20181481)
Supplement: Supplementary file 1 [file bsr20181481_Supp1.pdf]

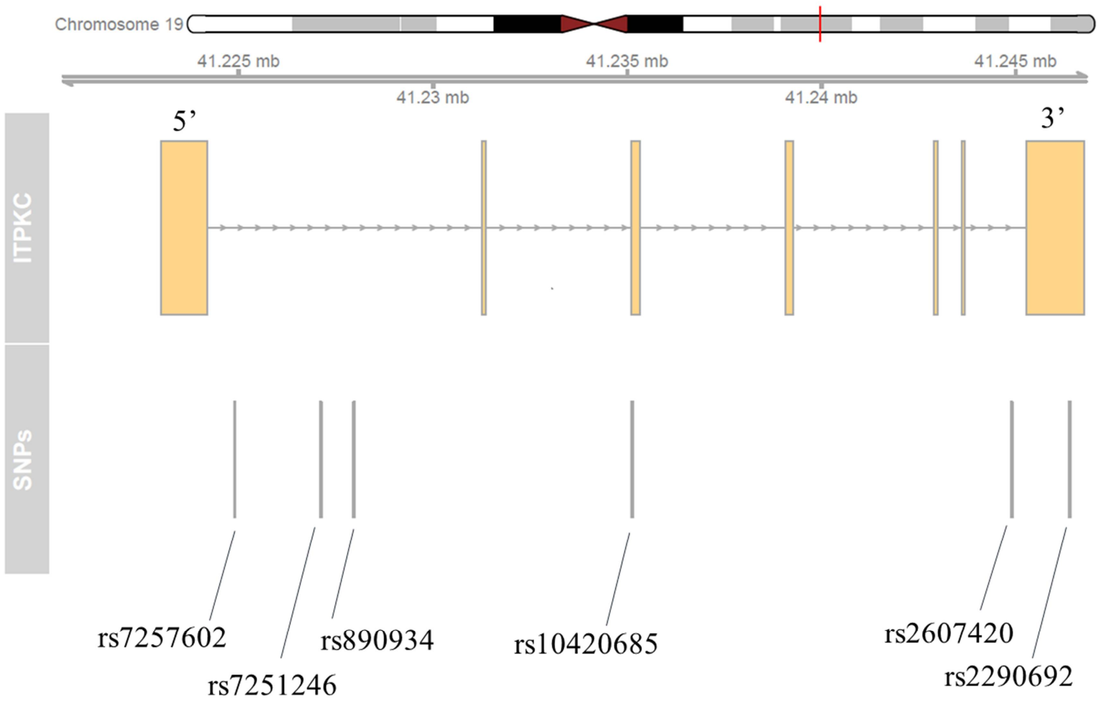

Supplementary Figure 1. Positions of the tag single nucleotide polymorphisms in the human *ITPKC* gene. Black and white boxes are coding and non-coding regions, respectively.

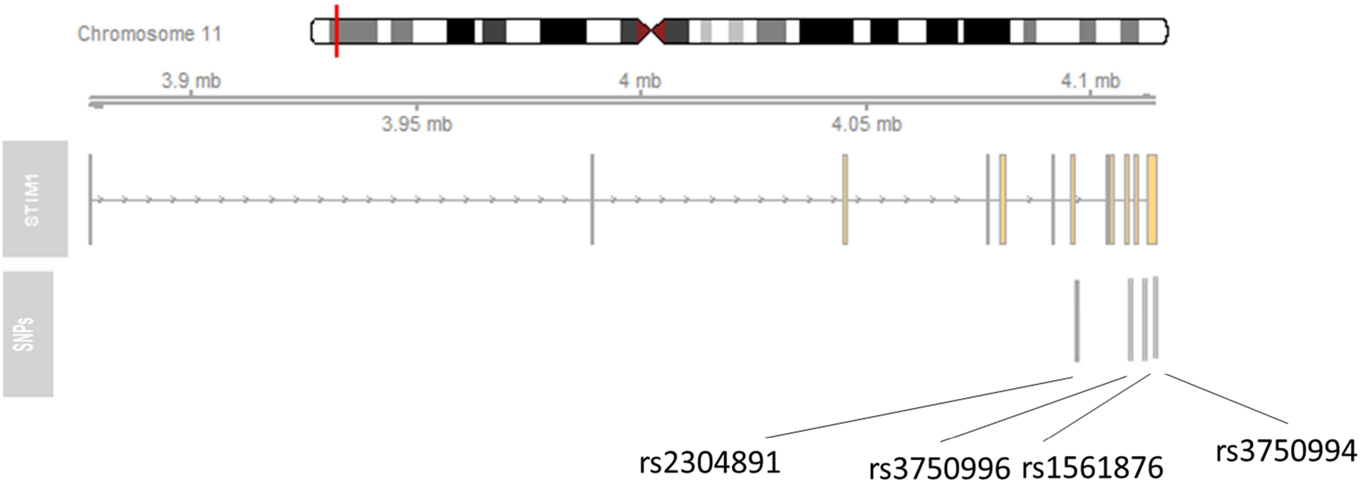

Supplementary Figure 2. Positions of the tag single nucleotide polymorphisms in the human *STIM1* gene. Black and white boxes are coding and non-coding regions, respectively.

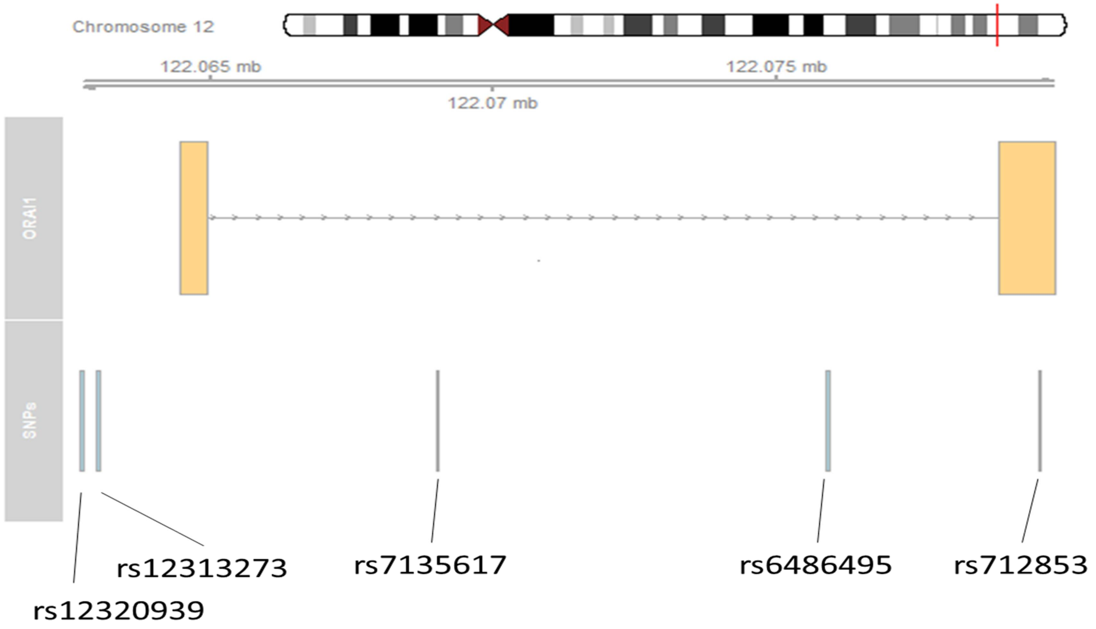

Supplementary Figure 3. Positions of the tag single nucleotide polymorphisms in the human *Orai1* gene. Black and white boxes are coding and non-coding regions, respectively.

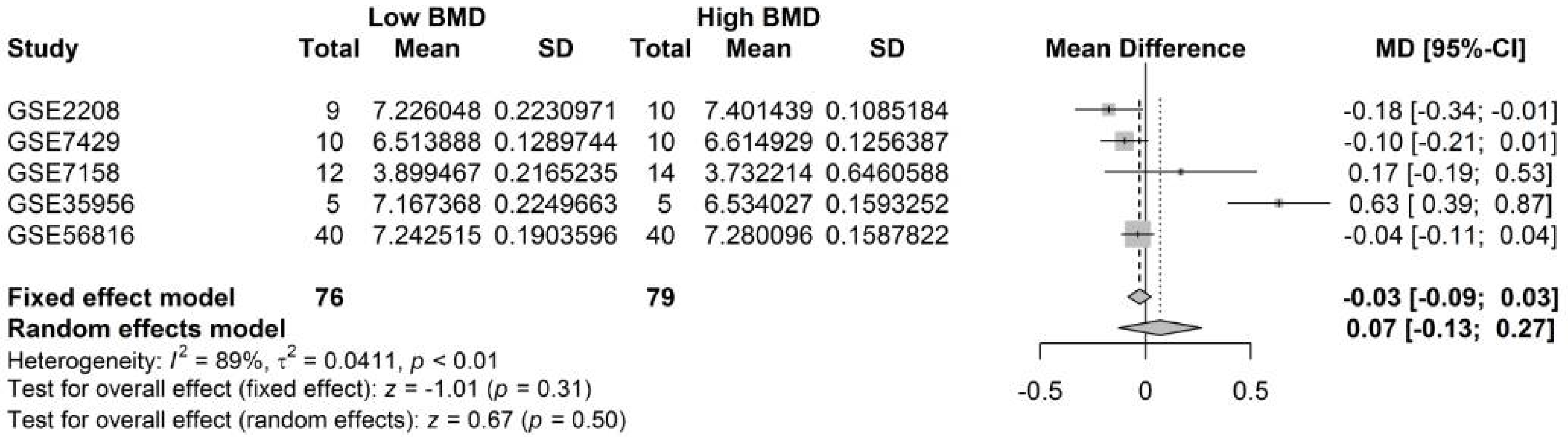

Supplementary Figure 4. Forest plot of *STIM1* expression profiling across five datasets

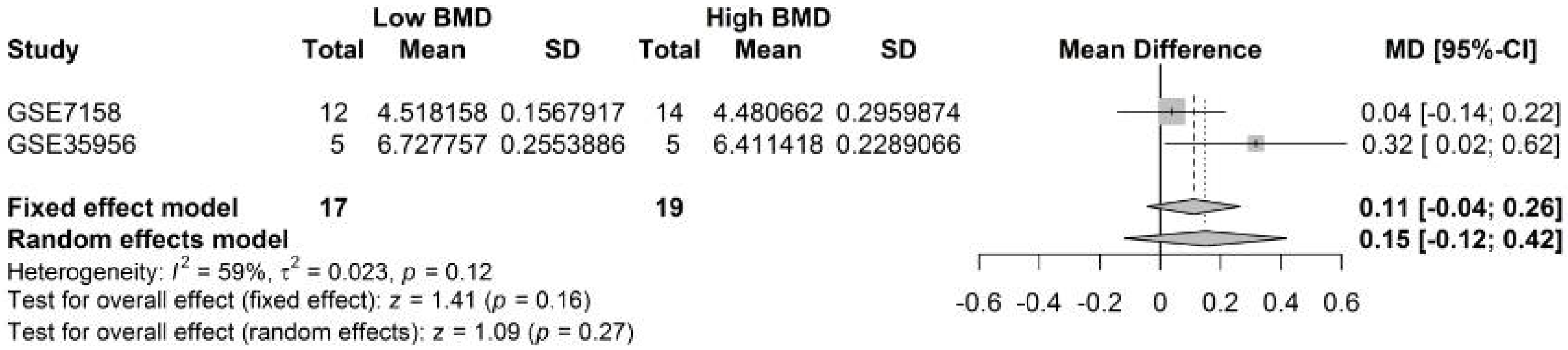

Supplementary Figure 5. Forest plot of *ORAI1* expression profiling across two datasets.

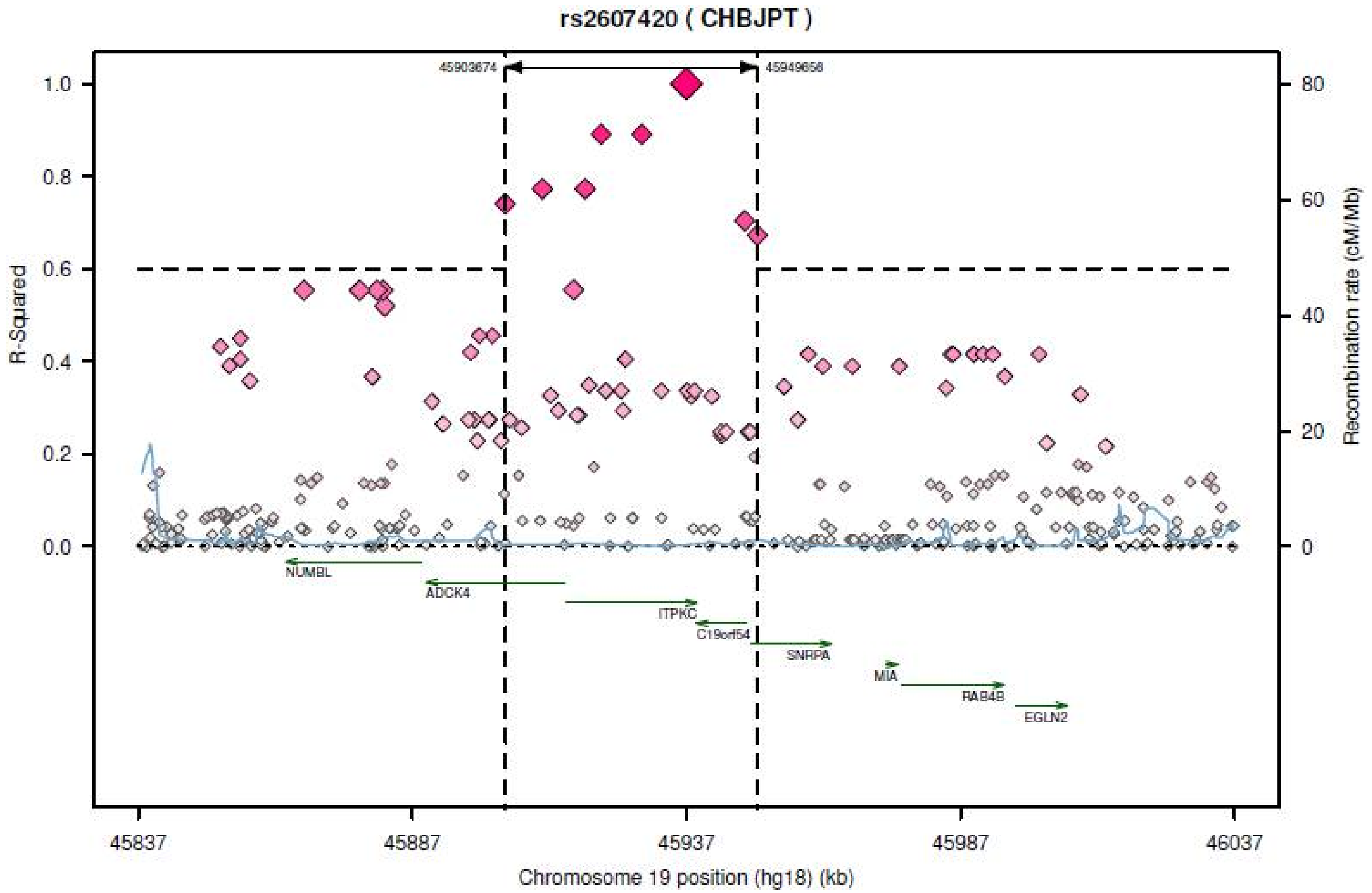

Supplementary Figure 6. Regional plot of rs2607420.

Supplementary Table 1. Basic characteristics of the tag single-nucleotide polymorphisms (SNPs) in *ITPKC*.

Gene	Chr	Position (hg38)	Variant	Ref	Alt	AFR	AMR	ASN	EUR	TWB	Our study	HWE p value
STIM1	11	4082294	rs2304891	A	G	0.10	0.54	0.48	0.56	0.44	0.41	0.55
STIM1	11	4091970	rs3750996	G	A	0.00	0.00	0.20	0.00	0.22	0.24	0.43
STIM1	11	4092165	rs1561876	A	G	0.28	0.80	0.79	0.88	0.27	0.29	0.94
STIM1	11	4092240	rs3750994	A	C	0.01	0.09	0.16	0.03	0.21	0.24	0.28
ORAI1	12	121624817	rs12320939	T	G	0.73	0.44	0.49	0.38	0.48	0.49	0.49
ORAI1	12	121625105	rs12313273	C	T	0.30	0.27	0.25	0.18	0.29	0.31	0.88
ORAI1	12	121631099	rs7135617	C	A	0.96	0.49	0.59	0.40	0.42	0.40	0.47
ORAI1	12	121638011	rs6486795	A	G	0.36	0.28	0.38	0.21	0.38	0.39	0.74
ORAI1	12	121641762	rs712853	T	C	0.62	0.22	0.36	0.24	0.32	0.32	0.24
ITPKC	19	40718993	rs7257602	A	G	0.24	0.56	0.47	0.45	0.43	0.47	0.02
ITPKC	19	40721201	rs7251246	T	C	0.22	0.56	0.51	0.49	0.48	0.49	0.26
ITPKC	19	40722063	rs890934	G	T	0.21	0.42	0.42	0.36	0.41	0.46	0.16
ITPKC	19	40729215	rs10420685	A	G	0.22	0.07	0.25	0.05	0.24	0.21	0.63
ITPKC	19	40738982	rs2607420	T	C	0.82	0.76	0.76	0.73	0.73	0.27	0.86
ITPKC	19	40740473	rs2290692	C	G	0.22	0.56	0.51	0.49	0.45	0.47	0.11

Ref: reference allele, Alt: Alternative allele. [*]Frequency of the Alt allele. AFR: African. AMR: Ad Mixed American. ASN: Asian. EUR: European. TWB: the Taiwan Biobank. HWE: Hardy-Weinberg equilibrium.

Supplementary Table 2**.** Association between single-nucleotide polymorphisms (SNPs) in *STIM1, ORAI1* and bone mineral density in the entire population.

			Lumbar Spine				Femoral Neck			
Gene	SNP	Genotype	n	Mean	SE	p-value	n	Mean	SE	p-value
STIM1	rs2304891	A/A	281	0.995	0.012	0.691	281	0.995	0.012	0.691
		G/A	364	0.969	0.010		364	0.969	0.010	
		G/G	131	1.024	0.017		131	1.024	0.017	
STIM1	rs3750996	G/G	458	0.984	0.009	0.128	458	0.984	0.009	0.128
		A/G	277	0.979	0.012		277	0.979	0.012	
		A/A	50	1.041	0.031		50	1.041	0.031	
STIM1	rs1561876	A/A	385	0.995	0.010	0.231	385	0.995	0.010	0.231
		G/A	298	0.967	0.011		298	0.967	0.011	
		G/G	61	1.003	0.028		61	1.003	0.028	
STIM1	rs3750994	A/A	450	0.990	0.010	0.740	450	0.990	0.010	0.740
		A/C	264	0.969	0.011		264	0.969	0.011	
		C/C	52	0.995	0.030		52	0.995	0.030	
ORAI1	rs12320939	T/T	213	0.988	0.013	0.971	213	0.988	0.013	0.971
		G/T	387	0.978	0.010		387	0.978	0.010	
		G/G	203	0.995	0.015		203	0.995	0.015	
ORAI1	rs12313273	C/C	379	0.996	0.010	0.823	379	0.996	0.010	0.823
		T/C	341	0.971	0.010		341	0.971	0.010	
		T/T	84	0.999	0.021		84	0.999	0.021	
ORAI1	rs7135617	C/C	288	0.986	0.011	0.483	288	0.986	0.011	0.483
		C/A	393	0.977	0.010		393	0.977	0.010	
		A/A	128	1.006	0.019		128	1.006	0.019	
ORAI1	rs6486795	A/A	299	0.996	0.012	0.960	299	0.996	0.012	0.960
		G/A	381	0.970	0.009		381	0.970	0.009	
		G/G	124	1.007	0.018		124	1.007	0.018	
ORAI1	rs712853	T/T	367	0.986	0.010	0.918	367	0.986	0.010	0.918

C/T	322	0.974	0.011		322	0.974	0.011
C/C	85	1.010	0.020		85	1.010	0.020

The *p* value was adjusted for age, sex and the body-mass index.

Supplementary Table 3. eQTL results from GTEx

SNP ID	Gencode ID (ENSG00000-)	Gene Symbol	P-Value	Effect Size	Tissue
rs2607420	188493.1	*C19orf54*	2.50E-12	-0.58	Nerve - tibial
	123815.7	*ADCK4*	9.90E-12	-0.39	Nerve - tibial
	188493.1	*C19orf54*	9.70E-11	-0.48	Thyroid
	188493.1	*C19orf54*	1.80E-09	-0.43	Artery - tibial
	188493.1	*C19orf54*	2.30E-09	-0.58	Pancreas
	188493.1	*C19orf54*	2.60E-09	-0.54	Artery - aorta
	188493.1	*C19orf54*	6.90E-09	-0.30	Stomach
	188493.1	*C19orf54*	1.00E-08	-0.37	Esophagus - muscularis
	86544.2	*ITPKC*	2.00E-08	-0.26	Skin - Sun Exposed (Lower leg)
	188493.1	*C19orf54*	7.10E-08	-0.60	Prostate
	188493.1	*C19orf54*	4.20E-07	-0.38	Adipose - subcutaneous
	188493.1	*C19orf54*	9.70E-07	-0.48	Spleen
	188493.1	*C19orf54*	1.70E-06	-0.27	Whole blood
